# Inflammation: an important parameter in the search of prostate cancer biomarkers

**DOI:** 10.1186/1477-5956-12-32

**Published:** 2014-06-09

**Authors:** Stefania Bergamini, Elisa Bellei, Luca Reggiani Bonetti, Emanuela Monari, Aurora Cuoghi, Francesco Borelli, Maria Chiara Sighinolfi, Giampaolo Bianchi, Tomris Ozben, Aldo Tomasi

**Affiliations:** 1 Department of Diagnostic Medicine, Clinic and Public Health, Proteomic Lab, University Hospital of Modena and Reggio Emilia, Via del Pozzo 71, 41124 Modena, Italy; 2 Section of Pathologic Anatomy, University Hospital of Modena and Reggio Emilia, Via del Pozzo 71, 41124 Modena, Italy; 3 Department of Biochemistry, Medical Faculty, Akdeniz University, Antalya, Turkey; 4 Department of Urology, University Hospital of Modena and Reggio Emilia, Via del Pozzo 71, 41124 Modena, Italy

**Keywords:** Inflammation, Prostate cancer biomarkers, Proteomics, SELDI-ToF-MS, 2-DE

## Abstract

**Background:**

A more specific and early diagnostics for prostate cancer (PCa) is highly desirable. In this study, being inflammation the focus of our effort, serum protein profiles were analyzed in order to investigate if this parameter could interfere with the search of discriminating proteins between PCa and benign prostatic hyperplasia (BPH).

**Methods:**

Patients with clinical suspect of PCa and candidates for trans-rectal ultrasound guided prostate biopsy (TRUS) were enrolled. Histological specimens were examined in order to grade and classify the tumor, identify BPH and detect inflammation. Surface Enhanced Laser Desorption/Ionization-Time of Flight-Mass Spectrometry (SELDI-ToF-MS) and two-dimensional gel electrophoresis (2-DE) coupled with Liquid Chromatography-MS/MS (LC-MS/MS) were used to analyze immuno-depleted serum samples from patients with PCa and BPH.

**Results:**

The comparison between PCa (with and without inflammation) and BPH (with and without inflammation) serum samples by SELDI-ToF-MS analysis did not show differences in protein expression, while changes were only observed when the concomitant presence of inflammation was taken into consideration. In fact, when samples with histological sign of inflammation were excluded, 20 significantly different protein peaks were detected. Subsequent comparisons (PCa with inflammation *vs* PCa without inflammation, and BPH with inflammation *vs* BPH without inflammation) showed that 16 proteins appeared to be modified in the presence of inflammation, while 4 protein peaks were not modified. With 2-DE analysis, comparing PCa without inflammation *vs* PCa with inflammation, and BPH without inflammation *vs* the same condition in the presence of inflammation, were identified 29 and 25 differentially expressed protein spots, respectively. Excluding samples with inflammation the comparison between PCa *vs* BPH showed 9 unique PCa proteins, 4 of which overlapped with those previously identified in the presence of inflammation, while other 2 were new proteins, not identified in our previous comparisons.

**Conclusions:**

The present study indicates that inflammation might be a confounding parameter during the proteomic research of candidate biomarkers of PCa. These results indicate that some possible biomarker-candidate proteins are strongly influenced by the presence of inflammation, hence only a well-selected protein pattern should be considered for potential marker of PCa.

## Background

Despite the improvements in clinical and surgical practice, prostate cancer (PCa) remains one of the most widespread cancers in males, with an unchanged mortality rate [1-4].

The serum marker currently used for the diagnosis of PCa is the prostate-specific antigen (PSA), which is not particularly reliable, having a predictive value estimated at 25%-35% in the range of 2.6 – 10 ng/mL [2]. Furthermore, benign conditions such as prostatitis and benign prostatic hyperplasia (BPH) can lead to an increase in PSA levels causing false positive [5,6]. With the aim of improving accuracy in the detection, monitoring and distinction between benign conditions and PCa, it is imperative to identify new and reliable molecular targets.

In recent years, proteomic techniques have achieved a rapid evolution, due to innovative experimental approaches and improvements in sensitivity, resolution and accuracy of the mass analysers. Several proteomic studies have been carried out on serum [7,8], urine [9], biopsy tissue [10] and cell lines [11,12], with the purpose of identifying promising targets for the early detection of PCa. Unfortunately, the majority of the candidate biomarkers are still awaiting validation [13,14]. Other studies have been performed in the attempt to discriminate PCa from BPH. Adam and co-workers used the protein profiling technology approach coupled with an artificial intelligence data analysis algorithm to distinguish PCa from non-cancer forms [15]. In another study, Ornstein et al. reported serum proteomic patterns obtained by Surface Enhanced Laser Desorption/Ionization-Time of Flight-Mass Spectrometry (SELDI-ToF-MS) analysis to discriminate PCa from BPH when PSA level is between 2.5 and 15 ng/mL, with the aim to decrease unnecessary prostate biopsies [16]. More recently, Ummanni et al. examined biopsy samples from BPH and PCa patients using two-dimensional gel electrophoresis (2-DE) followed by Matrix Assisted Laser Desorption/Ionization-Time of Flight-Mass Spectrometry analysis (MALDI-ToF-MS), to identify potential biomarkers that might differentiate the two clinical events [17].

In this study, in order to identify distinctive protein profiles able to unquestionably discriminate patients with a benign prostate condition from those with a malignant situation, the serum protein expression of PCa and BPH was investigated by proteomics.

Differently from previous publications, the benign states were considered *vs* the pathological ones focusing on the co-existence of inflammation, since emergent research underline a tight link between chronic inflammation and endothelial activation in both PCa and BPH [18-20].

Cancer and inflammation are closely linked, so much that cancer patients show both local and systemic changes in inflammatory parameters. In some cancer types, inflammatory conditions are present before a malignant change occurs; otherwise, in different type of cancer, an oncogenic alteration generates an inflammatory microenvironment that induces the development of tumors [21]. In PCa and BPH conditions, inflammation is frequently evident in prostate biopsies, radical prostatectomy specimens and tissue resected for the treatment of BPH. For this reason, the inflammatory injury, and consequently its chemical mediators and protein products, should be taken into account in proteomic studies aimed to identify PCa biomarkers.

In our study, serum samples were depleted of high-abundant proteins by immuno-chromatography and the depleted samples were analysed by SELDI-ToF-MS. This is a sensitive proteomic technique that analyses proteins on large scale in a relatively short time and therefore it is of help for the preliminary screening of complex samples and for biomarkers search. Subsequently, samples were analysed by 2-DE coupled with LC-MS/MS, in order to precisely identify relevant proteins.

## Results and discussion

Cancer survival rates depend on the early detection of the disease: currently, PCa diagnosis is performed using digital rectal exploration (DRE), trans-rectal ultrasound guided prostate biopsy (TRUS), and by the measurement of serum PSA levels. PSA is a sensitive marker for the detection of PCa, however it is not cancer-specific; elevated serum PSA levels are also observed in benign enlargements of the prostate, such as BPH or prostatitis, and after biopsy [22].

In this study, the serum proteins associated with PCa were compared to BPH, in order to identify distinctive protein profiles able to unquestionably discriminate patients with a malignant situation from those with a benign prostate condition. The study was conducted taking into consideration that inflammation is an intrinsic component of the cancer and BPH.

Because high-abundance proteins present in serum can interfere with resolution and sensitivity of proteomics, by masking low concentration proteins, serum samples were depleted by immunoaffinity chromatography. This procedure reduces the complexity of serum samples and enriches low-abundance proteins; moreover, it offers the lowest co-depletion of untargeted proteins, proving to be the most advantageous depletion approach for serum preparation prior to proteomic studies [23]*.*

The analysis was first carried out by SELDI-ToF-MS using the H50 ProteinChip surface, irrespective of the presence of inflammation. Under this condition, no differential expression of protein peaks was evident between PCa samples (n = 31) and BPH (n = 30).

Since inflammation is often associated to both PCa and BPH, its influence on the serum protein profile was tested. Therefore, after exclusion of samples with histological signs of inflammation, protein spectra from patients with PCa were compared to BPH (10 and 11 patients respectively).

This comparison showed evident differences in the protein profile, leading to the identification of 20 differentially expressed protein peaks; in particular, 5 peaks resulted increased (m/z 2325, 2348, 2373, 2581, 3104) and 15 peaks decreased (m/z 6624, 6837, 9352, 9922, 13775, 14031, 14106, 14473, 14763, 22668, 28052, 28242, 29018, 45350, 56390) in PCa compared to BPH (Table 1).

**Table 1:** **Differentially expressed peaks in PCa *vs *** **BPH excluding samples with inflammation detected by SELDI-ToF-MS**

**Peak**	**m/z**	**PCa intensity peak**	**BPH intensity peak**	***t*-test p-value**
**Increased**				
1	2325	4.29	1.32	0.002
2	2348	3.97	1.18	0.006
3	2373	3.27	1.10	0.005
4	2581	1.34	0.33	0.002
5	3104	2.19	0.88	0.007
**Decreased**				
1	6624	17.63	24.54	0.037
2	6837	2.37	3.19	0.010
3	9352	1.84	2.38	0.033
4	9922	0.44	0.66	0.048
5	13775	1.21	1.67	0.049
6	14031	2.76	4.98	0.001
7	14106	1.67	2.66	0.005
8	14473	0.55	0.85	0.0003
9	14763	0.57	0.76	0.002
10	22668	0.06	0.10	0.003
11	28052	2.05	3.90	0.003
12	28242	1.42	2.33	0.011
13	29018	0.48	0.93	0.003
14	45350	0.78	1.23	0.002
15	56390	0.84	1.32	0.026

Statistical analysis was performed by Student’s t test. The table shows the p-values statistically significant (p ≤ 0.05).

The specific protein expression of PCa with inflammation *vs* the same condition in absence of inflammation were subsequently compared. In this case, 9 protein peaks differentially expressed were detected: 4 peaks were increased (m/z 9352, 9922, 21739, 29018) and 5 peaks were decreased (m/z 2325, 2348, 3104, 3215, 17471) in the presence of inflammation. Notably, 6 protein peaks coincided with 6 of the 20 peaks differentially expressed in the comparison between PCa and BPH in the absence of inflammation (Table 2, peaks in italic).

**Table 2:** **Differentially expressed peaks in PCa with inflammation *vs *** **PCa without inflammation detected by SELDI-ToF-MS**

**Peak**	**m/z**	**PCa with inflammation intensity peak**	**PCa without inflammation intensity peak**	***t*-test p-value**
**Increased**				
1	*9352*	2.26	1.84	0.050
2	*9922*	0.64	0.45	0.040
3	21739	0.08	0.05	0.019
4	*29018*	0.75	0.49	0.043
**Decreased**				
1	*2325*	2.51	4.29	0.025
2	*2348*	2.28	3.97	0.044
3	*3104*	1.24	2.19	0.025
4	3215	1.49	1.96	0.024
5	17471	2.67	3.25	0.047

Statistical analysis was performed by Student’s *t* test. The table shows the p-values statistically significant (p ≤ 0.05). Protein peaks in italic: protein peaks found also in the comparison between PCa and BPH excluding samples with inflammation (Table 1).

The same approach was applied to BPH and 15 peaks were found to be differentially expressed when BPH samples with and without inflammation were compared: 5 increased (m/z 2325, 2348, 2373, 2581, 3104) and 10 decreased (m/z 6433, 6624, 6837, 9352, 14031, 14106, 14473, 22668, 28052, 45350) in the presence of inflammation. Particularly, all peaks differently expressed, except one (m/z 6433), overlapped with peaks found also in the comparison between PCa and BPH in the absence of inflammation (Table 3, peaks in square brackets).

**Table 3:** **Differentially expressed peaks in BPH with inflammation *vs *** **BPH without inflammation detected by SELDI-ToF-MS**

**Peak**	**m/z**	**BPH with inflammation intensity peak**	**BPH without inflammation intensity peak**	***t*-test p-value**
**Increased**				
1	[2325]	3.28	1.32	0.016
2	[2348]	3.34	1.84	0.013
3	[2373]	2.93	1.10	0.009
4	[2581]	1.06	0.33	0.007
5	[3104]	1.74	0.88	0.037
**Decreased**				
1	6433	9.65	12.61	0.009
2	[6624]	18.39	24.54	0.017
3	[6837]	2.51	3.19	0.018
4	[9352]	1.92	2.38	0.037
5	[14031]	3.49	4.98	0.012
6	[14106]	2.00	2.66	0.036
7	[14473]	0.68	0.85	0.033
8	[22668]	0.07	0.10	0.011
9	[28052]	2.82	3.90	0.033
10	[45350]	0.94	1.23	0.037

Statistical analysis performed by Student’s *t* test. The table shows the p-values statistically significant (p ≤ 0.05). Protein peaks in square: protein peaks found also in the comparison between PCa and BPH excluding samples with inflammation (Table 1).

**Figure 1:**
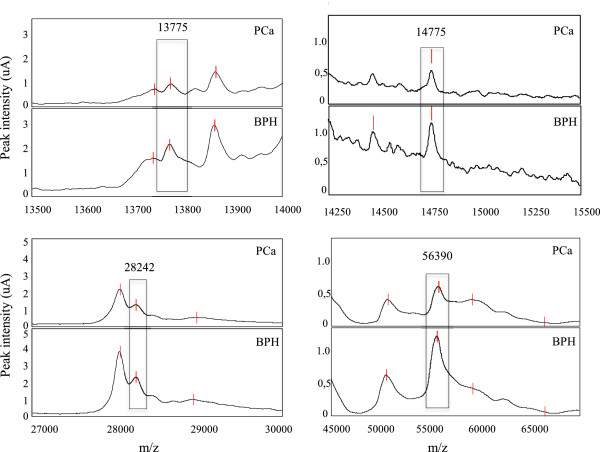
Representative spectra obtained by SELDI-ToF-MS analysis concerning the 4 statistically significant peaks detected with H50 ProteinChip Array.

In summary, SELDI-ToF-MS analysis demonstrated that, in the absence of inflammation, 20 different protein peaks are expressed in PCa in respect to BPH. Of these, only 4 peaks, highlighted in Table 4 (in bold) and shown in Figure 1, could potentially differentiate PCa from BPH, since their expression is not altered by the presence of inflammation. The remaining 16 peaks (also found differentially expressed in presence of inflammation) seem to be strongly related to inflammation, hence they can not be used as markers of PCa (Table 4). The inflammatory process therefore appears to be a limiting factor in the search of biomarkers able to discriminate PCa from BPH and it has not be underrated, since it represents a key mechanism in the development and progression of both diseases [24]. Inflammation is often observed in tumors, with immune cell infiltration and activated stroma. For this reason, cancer patients frequently present changes in various systemic parameters, comprising alterations in the level of serum inflammatory cytokines, acute-phase proteins and total albumin [25]. In fact, inflammation generates not only cancer-promoting microenvironment changes, but also systemic alterations that promote cancer progression [26]. Despite the evidence that inflammation is an intrinsic component of cancer, this fundamental aspect is often ignored in biomarker research studies.

**Table 4:** **Comparison of peaks intensities differentially expressed in PCa *vs *** **BPH detected by SELDI-ToF-MS**

**Peak**	**m/z**	**Intensity peak**			
		**PCA (n = 31)**		**BPH (n = 30)**	
		**Inflammation**		**Inflammation**	
		**Absent (n = 10)**	**Present (n = 21)**	**Absent (n = 11)**	**Present (n = 19)**
1	2325	4.30	2.51	1.32	3.28
2	2348	3.97	2.28	1.84	3.34
3	2373	3.28	1.95*	1.10	2.93
4	2581	1.34	0.85*	0.33	1.06
5	3104	2.20	1.24	0.88	1.74
6	6624	17.63	18.93*	24.54	18.39
7	6837	2.37	2.54*	3.19	2.51
8	9352	1.84	2.26	2.38	1.92
9	9922	0.44	0.64	0.66	0.52*
10	**13775**	1.21	1.58*	1.67	1.57*
11	14031	2.76	3.74*	4.98	3.49
12	14106	1.67	2.14*	2.66	2.00
13	14473	0.55	0.73*	0.85	0.68
14	**14763**	0.57	0.66*	0.76	0.71*
15	22668	0.06	0.08*	0.10	0.07
16	28052	2.05	3.06*	3.90	2.82
17	**28242**	1.42	1.88*	2.33	1.81*
18	29018	0.48	0.75	0.93	0.68*
19	45350	0.78	1.00*	1.23	0.94
20	**56390**	0.84	1.24*	1.32	1.09*

Protein peaks in bold: protein peaks discriminating PCa from BPH.

*Differences not statistically significant.

The indications reported above and the preliminary results obtained in the present study by SELDI-ToF-MS analysis, suggested that inflammation could be a confounding factor in the identification of protein profiles able to discriminate between PCa and BPH. Afterward, to verify this supposition, proteomic analysis was performed by 2-DE coupled with MS.

**Figure 2:**
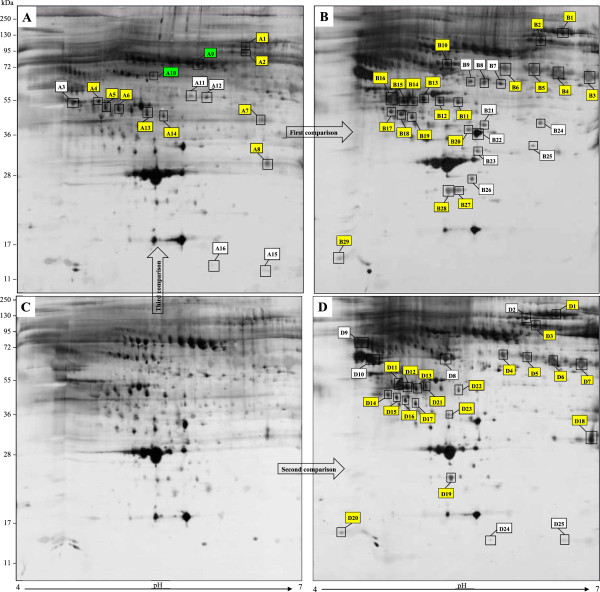
**Bi-dimensional proteome maps of serum samples from PCa without (A) and with inflammation (B), and BPH in absence (C) and presence of inflammation (D).** Proteins were resolved by IEF over the pH range 4–7, followed by 8-16% gradient gel and visualized by Silver staining. Significant differentially expressed proteins are marked with alphanumeric labels, corresponding to those listed in Tables 5, 6 and 7. Yellow tags indicate the overlapped proteins detected in presence of inflammation in both PCa **(B)** and BPH **(D)** conditions. Some of these proteins were also revealed in PCa in absence of inflammation (**A**, third comparison). Additionally, in this latter situation, green labels represent proteins not previously identified in the first and second comparisons, namely in presence of inflammation.

Representative 2-D gels obtained from depleted serum samples are reported in Figure 2. Inflammation-free PCa *vs* PCa with inflammation were first compared (first comparison); then, BPH was considered in the absence or presence of inflammation (second comparison), and finally the two conditions were compared with the exclusion of inflammation (third comparison). The differentially expressed protein spots are marked in the images by alphanumeric labels, that correspond to those reported in the first column of Tables 5, 6 and 7, respectively. The second column of Tables 5, 6 and 7 refers to the primary accession number, derived from the UniProt knowledge database, the third column provides the complete name of each identified protein and column 4 reports the theoretical molecular weight (MW). Column 5 shows the highest ion scores obtained with MASCOT search engine, expressed as the probability that the observed match between the experimental data and the database sequence could be due to a random event. Column 6 indicates the total number of peptides that matched the identified proteins and the significant matches, while column 7 reports the total number of sequences and the number of significant sequences. Finally, the last column reports the protein expression change, indicated by arrows.

**Table 5:** **Differentially expressed proteins in PCa without inflammation *vs *** **PCa with inflammation (UniProtKB database)**

**Spot n°.**	**Acc. n°.**	**Protein full name**	**Mass (Da)**	**Score**	**N° matchs/signif. matchs**	**N° seq./signif. seq.**	**Expression change**
B1	P00751	*Complement factor B*	86847	445	212/60	33/19	↑
B2	P00734	*Prothrombin*	71475	455	75/39	15/7	↑
B3	P02749	*Beta-2-glycoprotein 1*	39584	458	81/42	17/8	↑
B4	P02749	*Beta-2-glycoprotein 1*	39584	288	69/32	14/12	↑
B5	P02749	*Beta-2-glycoprotein 1*	39584	122	55/17	11/8	↑
B6	P02749	*Beta-2-glycoprotein 1*	39584	35	28/4	6/3	↑
B7	P36955	Pigment epithelium-derived factor	46454	142	46/13	11/8	↑
B8	P36955	Pigment epithelium-derived factor	46454	65	42/12	13/5	↑
B9	P36955	Pigment epithelium-derived factor	46454	51	35/8	13/7	↑
B10	Q14624	*Inter-alpha-trypsin inhibitor heavy chain H4*	103521	375	152/47	30/13	↑
B11	P00738	*Haptoglobin*	45861	110	83/21	17/7	↑
B12	P00738	*Haptoglobin*	45861	236	99/28	18/8	↑
B13	P00738	*Haptoglobin*	45861	138	97/16	18/6	↑
B14	P25311	*Zinc-alpha-2-glycoprotein*	34465	104	24/9	10/4	↑
B15	P01024	*Complement C3 (fragment)*	188569	104	49/8	21/4	↑
B16	P01024	*Complement C3 (fragment)*	188569	2354	365/180	38/27	↑
B17	P10909	*Clusterin*	53031	241	57/16	10/4	↑
B18	P10909	*Clusterin*	53031	138	31/12	7/3	↑
B19	P10909	*Clusterin*	53031	202	55/22	11/7	↑
B20	Q14624	*Inter-alpha-trypsin inhibitor heavy chain H4*	103521	41	13/3	2/2	↑
B21	P02649	Apolipoprotein E	36246	398	125/43	27/13	↑
B22	P02766	Transthyretin	15991	1086	165/71	16/11	↑
B23	P02743	Serum amyloid P-component	25485	304	43/20	8/6	↑
B24	O75636	Ficolin-3	33395	205	71/24	12/7	↑
B25	P36980	Complement factor H-related protein 2	31543	70	25/8	6/4	↑
B26	O95455	Apolipoprotein M	21582	49	22/3	7/3	↑
B27	P02753	*Retinol binding protein 4*	23337	230	42/15	7/5	↑
B28	P02753	*Retinol binding protein 4*	23337	1071	154/74	9/8	↑
B29	P02656	*Apolipoprotein C-III*	10846	84	10/4	2/4	↓

Protein names in italic: proteins found also in the comparison between BPH without inflammation and BPH with inflammation (Table 6).

**Table 6:** **Differentially expressed proteins in BPH without inflammation *vs *** **BPH with inflammation**

**Spot n°.**	**Acc. n°.**	**Protein full name**	**Mass (Da)**	**Score**	**N° matchs/signif. matchs**	**N° seq./signif. seq.**	**Expression change**
D1	P00751	*Complement factor B*	86847	501	196/57	34/15	↑
D2	P06396	Gelsolin	86043	301	141/37	25/14	↑
D3	P00734	*Prothrombin*	71475	168	42/14	12/5	↑
D4	P02749	*Beta-2-glycoprotein 1*	39584	39	15/2	5/2	↑
D5	P02749	*Beta-2-glycoprotein 1*	39584	41	32/15	7/4	↑
D6	P02749	*Beta-2-glycoprotein 1*	39584	331	72/30	13/9	↑
D7	P02749	*Beta-2-glycoprotein 1*	39584	204	70/33	13/7	↑
D8	P02774	Vitamin-D binding protein	54526	1679	283/149	32/28	↑
D9	P01011	Alpha-1-antichymotrypsin	47792	324	54/24	11/8	↑
D10	P02765	Alpha-2-HS-glycoprotein	40098	273	96/38	11/9	↑
D11	P01024	*Complement C3 (fragment)*	188569	700	210/73	33/16	↑
D12	P01024	*Complement C3 (fragment)*	188569	1500	300/126	42/23	↑
D13	P25311	*Zinc-alpha-2-glycoprotein*	34465	66	15/6	6/3	↑
D14	P10909	*Clusterin*	53031	83	21/4	8/2	↑
D15	P10909	*Clusterin*	53031	245	42/14	8/4	↑
D16	P10909	*Clusterin*	53031	159	39/14	8/6	↑
D17	P10909	*Clusterin*	53031	174	25/11	6/3	↑
D18	P01024	*Complement C3 fragment*	188569	834	119/57	16/10	↑
D19	P02753	*Retinol binding protein 4*	23337	585	134/64	9/9	↑
D20	P02656	*Apolipoprotein C-III*	10846	231	7/7	2/2	↑
D21	P00738	*Haptoglobin*	45861	524	102/43	15/10	↓
D22	P00738	*Haptoglobin*	45861	348	90/34	17/9	↓
D23	Q14624	*Inter-alpha-trypsin inhibitor heavy chain H4*	103521	222	30/19	7/6	↓
D24	P0DJI8	Serum amyloid A-1 protein	13581	269	26/13	10/5	↓
D25	P0DJI8	Serum amyloid A-1 protein	13581	360	29/16	10/5	↓

Protein names in italic: proteins found also in the comparison between PCa without inflammation and PCa with inflammation (Table 5).

**Table 7:** **Proteins differentially expressed in the absence of inflammation in PCa *vs *** **BPH**

**Spot n°.**	**Acc. n°.**	**Protein full name**	**Mass (Da)**	**Score**	**N° matchs/signif. matchs**	**N° seq./signif. seq.**	**Expression change**
A1	P00734	*Prothrombin*	71475	122	29/11	8/5	↑
A2	P00734	*Prothrombin*	71475	31	17/2	6/2	↑
A3	P0C0L5	Complement C4-B (fragment)	194170	2320	117/90	20/17	↑
A4	P01024	*Complement C3 (fragment)*	188569	1531	134/78	54/36	↑
A5	P01024	*Complement C3 (fragment)*	188569	209	27/12	15/6	↑
A6	P00738	*Zinc-alpha-2-glycoprotein*	45861	1037	95/61	20/17	↑
A7	P01024	*Complement C3 (fragment)*	188569	46	9/3	6/3	↑
A8	P01024	*Complement C3 (fragment)*	188569	69	24/8	7/5	↑
A9	P02790	**Hemopexin**	52385	1160	310/114	30/21	↓
A10	P01008	**Antithrombin-III**	53025	542	136/50	26/13	↓
A11	P36955	Pigment epithelium-derived factor	46454	166	33/15	10/8	↓
A12	P36955	Pigment epithelium-derived factor	46454	422	61/35	12/10	↓
A13	P00738	*Haptoglobin*	45861	437	52/31	8/8	↓
A14	P00738	*Haptoglobin*	45861	871	90/52	20/15	↓
A15	P0DJI8	Serum amyloid A-1 protein	13581	111	17/8	8/2	↓
A16	P0DJI8	Serum amyloid A-1 protein	13581	244	34/22	9/6	↓

Protein names in italic: proteins found also in the previous comparison in the presence of inflammation (Tables 5 and 6).

Protein names in bold: proteins not previously identified in presence of inflammation.

In the presence of inflammation, the first comparison showed 29 spots differentially expressed corresponding to 17 unique proteins (Table 5 and Figure 2B), while the second comparison showed 25 spots differentially expressed corresponding to 15 unique proteins (Table 6 and Figure 2D).

Ten unique proteins, corresponding to 20 and 19 spots in the first and second comparison respectively, were found to be common to both PCa and BPH in the presence of inflammation (yellow labels in Figure 2B and in 2D, respectively). Seven of these proteins resulted increased in both conditions: Complement factor B, Prothrombin, Beta-2-glycoprotein 1, Complement C3 fragment, Zinc-alpha-2-glycoprotein, Clusterin and Retinol binding protein. Apolipoprotein CIII appeared decreased in PCa and increased in BPH, while Inter-alpha-trypsin inhibitor heavy chain and Haptoglobin resulted increased in PCa and decreased in BPH (Tables 5 and 6, proteins name in italic).

When the two conditions were compared in the absence of inflammation (third comparison), 9 unique proteins differentially expressed, corresponding to 16 spots, were found in PCa (Figure 2A and Table 7). Precisely, 4 resulted increased: Prothrombin, Complement C4-B, fragments of Complement C3 and Zinc-alpha-2-glycoprotein; while 5 were decreased: Hemopexin, Antithrombin-III, Pigment epithelium-derived factor, Haptoglobin and Serum Amyloid A-1 protein.

Serum Amyloid A-1 protein is an acute phase protein that is synthesized under the regulation of inflammatory cytokines during both acute and chronic inflammation [27]. In a recent study reported by Menschikowski et al., this protein is considered a circulating biomarker of inflammation during BPH development and PCa progression [28]. In our study, Serum Amyloid A-1 protein was found decreased in BPH without inflammation (Table 6) in accordance with the literature data, while in PCa patients this protein did not shown any changes.

As highlighted in Figure 2A (yellow labels) and in Table 7 (protein names in italic), in third comparison were found some proteins identified also in PCa and BPH in the presence of inflammation (probably inflammation-linked proteins), such as fragments of Complement C3, Prothrombin, Haptoglobin and Zinc-alpha-2-glycoprotein. This can be clearly explained since a certain degree of inflammation is always present in PCa.

The most interesting result observed in third comparison is the detection of 2 proteins, not identified in the previous comparisons, namely Hemopexin and Antithrombin-III, (Figure 2A, green labels and Table 7, protein names in bold).

Hemopexin (or β_1B_-glycoprotein) is a heme-binding serum protein with high carbohydrate content and immunoelectrophoretic identity. The most important physiological role of Hemopexin is to act as an antioxidant in case of heme overload, rather than to participate in iron metabolism [29]. In a recent work, Hemopexin N-glycan profile was indicated to be of diagnostic value in hepatocellular carcinoma patients [30].

Antithrombin-III is a member of the serpin family and functions as an inhibitor of thrombin and other enzymes involved in the clotting cascade; moreover, it has been demonstrated to possess a potent antiangiogenic activity and antitumor action [31]. Cao et al. have examined the expression of Antithrombin in benign and malignant prostate gland. They found that this protein was widely expressed in PCa, but was gradually lost in tumors with high Gleason grade [32]. A decrease in plasma levels of Antithrombin-III is also reported in patients with colon and ovarian cancer, especially in presence of metastasis [33], other than in PCa [34]. In our study, we found a significantly lower expression of Antithrombin-III in PCa than the BPH. Hence, the local anti-angiogenic activity of Antithrombin-III may be partially lost in advanced stages of PCa.

The comparison of the protein profile between PCa and BPH by 2-DE showed several differentially expressed proteins, the majority of which could be related to the inflammatory process and not to the pathological condition. These results confirm those obtained by SELDI-ToF-MS analysis although it is not possible to perform a direct correspondence between the two techniques because the analytical conditions are different (pre-analytical sample treatment, detection of proteins in different mass range, use of selective chromatographic surface with the SELDI-ToF-MS technology).

## Conclusions

This paper emphasizes the importance of considering inflammation in endeavours aimed to the discovery of specific markers capable to differentiate PCa from BPH. Using two different proteomic techniques we have clearly demonstrated that, in the presence of inflammation, the majority of the differentially expressed protein peaks detected by SELDI-ToF-MS and of protein spots revealed by 2-DE analysis can’t be considered discriminating markers of PCa. Therefore, the inflammatory process masks the detection of some proteins, which could be the real differential targets between the malignant and benign condition.

## Methods

### Study population

Ninety patients with clinical suspect of PCa (serum PSA elevation and/or palpable mass at DRE) and candidates for TRUS guided biopsy were enrolled. The recruitment was done at the Department of Urology, University Hospital of Modena and Reggio Emilia. Participants provided written informed consent and the local research Ethical Committee (Comitato Etico Provinciale di Modena) approved the study design.

The median patients age was 67 years (range 57–81). We excluded subjects with relevant systemic diseases or significant clinical events during the 6 months before the recruitment and patients that received hormonal treatment or radio-chemotherapy. All patients underwent a 12 months follow up.

### Histological examination and patients’ classification

All enrolled patients underwent to TRUS guided biopsy with a 16-G needle. A total of 12 samples (6 per side) were obtained from each biopsy. Each transrectal ultrasound performed included an assessment of prostatic diameter, the volume of the whole prostate, and the transition zone and capsular and seminal vesicle characteristics, as well as a morphological description of potential pathological features in the peripheral or transition zone. Each histological specimen was examined by two pathologists, with the aim to recognize conditions of PCa and BPH, besides the presence of inflammation. The histological criterion used to define inflammation was the presence of pathological infiltration of the prostatic tissue by inflammatory cells, evaluated by hematoxylin and eosin stain.

Radical prostatectomy was performed in cases of histological diagnosis of PCa at biopsy.

Histological examination recognized PCa (31 patients), BPH (30 patients), high-grade prostatic intraepithelial neoplasia (HGPIN, 13 patients), prostate adenoma (PA, 5 patients) and inflammation.

BPH was defined a non neoplastic increase of glandular and/or stromal components of the prostatic glands clinically characterized by enlargement of the gland, while PA was defined a prominent nodular proliferation of crowded benign small to medium-sized glands with inconspicuous nuclei.

Since the purpose of the present study was to detect differences in the serum proteomic profiles of PCa and BPH, all cases of HGPIN and PA were excluded. Furthermore, others 11 cases were not analyzed due to problems occurred during serum processing. Then, the analysis was performed on a total of 61 cases from the initial 90 patients enrolled. Clinical data of the patients analyzed are shown in Table 8.

**Table 8:** Clinical data of enrolled patients

	**PCa (n = 31)**	**BPH (n = 30)**
Median age (years)	67	68
PSA (range ng/mL)	0.20 – 25.00	0.80 – 34.36
Gleason score		
G < 7	14	/
G ≥ 7	17	/
Tumor clinical stage		
T1	5	/
T2	20	/
T3	6	/
Inflammation		
Absence	10	11
Presence	21	19

Tumors were stratified according to the TNM classification [35] and Gleason grading system [36]: 25 cases were organ-confined tumor (pT1-pT2) and 6 cases were non-organ confined tumor (pT3); 14 cases were graduated as Gleason score < 7 and 7 cases with Gleason score ≥ 7.

### Serum samples

Before prostatic biopsy, venous blood was collected into vacutainer serum separation tubes and allowed to clot at room temperature for 1 hour. Serum was separated by centrifugation at 2,000 x g for 10 min at 4°C. After the addition of a protease inhibitor cocktail (Sigma-Aldrich) to prevent protein enzymatic breakdown or modifications, samples were divided into aliquots and kept frozen at −80°C until use. A quality control sample (QC) was prepared by pooling an equal amount of serum from healthy donors. The QC sample was used to assess the reproducibility of each SELDI-ToF-MS experiment and as control for each obtained protein profile.

### Serum immunodepletion

The presence of highly abundant proteins can interfere with the resolution and sensitivity of the proteomic techniques used to analyse the serum profiles. For this reason, serum samples were depleted by immunoaffinity chromatography using a Multiple Affinity Removal System (MARS) column (4.6 mm ID × 100 mm, Agilent Technologies Inc., CA, USA) containing antibodies against the six most abundant serum proteins: albumin, IgG, IgA, transferrin, haptoglobin and alpha-1-antitrypsin. The depletion is expected to remove about 88-92% of the total protein content. Removal was performed according to the recommendations of the manufacturer. Briefly, 200 μL of diluted sample were injected in a Beckman System Gold HPLC (Beckman Coulter, Fullerton, CA, USA). The low-abundance protein fractions eluted were aliquoted and stored at −20°C until analysis. The QC was also depleted following the same procedure.

### SELDI-ToF-MS analysis

A SELDI-ToF mass spectrometer Series 4000 (Bio-Rad Laboratories Inc., Hercules, CA, USA) was used to analyse the depleted serum samples. After some tests, the H50 (reverse-phase) ProteinChip Array, a surface able to selectively bind proteins with hydrophobic residues, gave the best serum profiles in terms of proteins number and resolution, so was selected for our experiments.

The analysis was performed as described in Monari et al. [37]. Shortly, depleted serum sample (20 μL) was mixed with ProteinChip binding buffer (110 μL) containing 10% acetonitrile (ACN) and 0.1% trifluoroacetic acid (TFA), and loaded onto pre-equilibrated H50 ProteinChip Array spot surfaces. After incubation, saturated sinapinic acid solution was applied to each spot and samples were analyzed with two different reading protocols optimized for low and high MW (laser energy 2500 nJ and 5000 nJ, respectively). The protein mass spectra were generated using an average of 901 laser shot, and the “All-in-one protein standard” (Bio-Rad) was used to generate a protein standard spectrum for mass accuracy calibration, for both reading protocols.

ProteinChip Data Manager 3.0 software (Bio-Rad) was employed for statistical analysis; the spectra were calibrated, baseline subtracted, mass aligned and normalized by total ion current in the range of 2–30 kDa and 30–100 kDa for low and high MW, respectively. Poor quality spectra with a normalization factor greater than twice the median value were excluded. Supervised clustering was performed using the following settings: 5 times signal-to-noise (S/N) ratio, 3 times S/N valley depth, 20% min peak threshold in the first pass for peaks identification, and 2 times S/N ratio on the second pass for cluster completion.

To asses reproducibility the coefficient of variation (% CV) of peaks obtained from QC sample replicates were used. The pooled% CV mean was 19.08% indicating that no analytical bias was present during the experiment.

After clusters identification, Student *t*-tests were performed and a p-value ≤ 0.05 was accepted as statistically significant.

### Two-dimensional gel electrophoresis

The immunodepleted serum samples, precisely the low-abundance protein fractions eluted, were first buffer-exchanged using 20 mm Tris–HCl, pH 7.4, by 5 kDa MW cut-off spin concentrators (Agilent Technologies). The samples were subjected to 3 cycles of buffer addition, with centrifugation at 7,500 x g at 10°C for 20 min every time.

Protein concentration was determined by the Bradford method [38], using the Protein Assay Dye Reagent (Bio-Rad). Eighty micrograms of proteins were diluted to 300 μL with a rehydration buffer composed of 6 M urea, 2 M thiourea, 4% CHAPS, 25 mM dithiothreitol (DTT), 0.2% ampholytes, and loaded onto 17 cm immobilized pH-gradient (IPG) strips, pH range 4–7 (Ready Strip™, Bio-Rad). Proteins were separated in first dimension through isoelectric focusing at 20°C, by an initial step of rehydration at 50 V for 12 h, followed by a second step at 250 V for 15 min, ramping up to 10.000 V for 3 h and finally focusing to reach 75.000 V-h.

Subsequently, the strips were equilibrated by incubation for 15 min at room temperature, first with 1% DTT and then with 2.5% iodoacetamide, both dissolved in equilibration buffer containing 6 M urea, 2% sodium-dodecyl-sulphate (SDS), 30% glycerol, 50 mM Tris–HCl pH 8.8 and trace of bromophenol blue. Second dimension separation was achieved by SDS-PAGE (SDS-polyacrylamide gel electrophoresis), using 8-16% acrylamide gradient gel and a running buffer composed of 192 mM glycine, 0.1% SDS and 25 mM Tris–HCl, pH 8.3. The equilibrated strips were embedded into a solution of 0.5% agarose on the top of the gel and electrophoresis was performed at 80 mA/gel for the first 30 min, then the voltage increased to 500 V until the dye font reached the bottom of the gel. After 2-DE, proteins were visualized by a Silver nitrate staining protocol, as previously reported in detail [39].

Then, the gels were acquired using a GS-800 calibrated densitometer (Bio-Rad) and the gel images were exported to the PDQuest 2-D Image Analysis software, version 7.3.1. (Bio-Rad). This software compares 2-DE gel images, identifying differential protein expression by the detection of increase or decrease protein spot on the basis of their staining intensities.

In the present study, the differentially expressed protein spots were excised and “in-gel” trypsin digested, as previously fully described [39].

### Protein identification by LC MS/MS

Obtained peptides were vacuum concentrated and then were dissolved in 10 μL of Buffer A, composed of 3% ACN with 0.1% Formic Acid (FA). Digested peptides (4 μL) were analyzed by the 6520 Accurate-Mass ESI-Q-ToF coupled with a 1200 Nano HPLC-Chip microfluidic device (Agilent Technologies). Sample separation was performed as previously described figure In brief, the samples were loaded from the autosampler into the Chip enrichment column (Zorbax C18, 4 mm × 5 μm i.d., Agilent Technologies) by a capillary pump, with a loading flow of 4 μL/min using Buffer A. Nitrogen was used as the nebulising gas. A separation column (Zorbax C18, 43 mm × 75 μm i.d., Agilent Technologies) was used for peptide separation, setting the analytical flow rate at 0.4 μL/min. Elution was obtained with Buffer B (97% ACN, 0.1% FA). Total run time was 40 min.

Protein-identification peak lists were generated using Mascot server (Matrix Science, UK. Mascot 2.4). SwissProt protein database (SwissProt 2013_04) was selected, specifying the following parameters: *Homo sapiens* taxonomy, parent ion tolerance ± 20 ppm, MS/MS error tolerance ± 0.1 Da, alkylated cysteine as fixed modification, oxidized methionine as variable modification, and two potential missed trypsin cleavages. Proteins were considered identified with at least 2 unique peptides.

## Abbreviations

PCa: Prostate cancer; PSA: Prostate-specific antigen; BPH: Benign prostatic hyperplasia; SELDI-ToF-MS: Surface enhanced laser desorption/ionization - time of flight - mass spectrometry; 2-DE: Two-dimensional gel electrophoresis; LC-MS/MS: Liquid chromatography MS/MS; DRE: Digital rectal exploration; TRUS: Trans-rectal ultrasound guided prostate biopsy; MW: Molecular weight; HGPIN: High-grade prostatic intraepithelial neoplasia; PA: Prostate adenoma; QC: Quality control; S/N: Signal-to-noise; CV: Coefficient of variation; DTT: Dithiothreitol; SDS: Sodium-dodecyl-sulphate; ACN: Acetonitrile; FA: Formic acid.

## Competing interests

The authors declare that they have no competing interests.

## Authors’ contributions

SB designed the study, performed immunodepletion of serum and drafted the manuscript. LRB performed histological analysis for each histological specimen. EM carried out SELDI-TOF-MS analysis and performed statistical analysis. EB performed bi-dimensional gel electrophoresis and helped to draft the manuscript. AC performed protein identification by mass spectrometry and helped in SELDI-TOF-MS experiments. TO and AT provided useful advices to improve the study and revised the manuscript. FB participated in samples collection and carried out samples preparation for mass spectrometry analysis. MCS was responsible for patient’s recruitment and samples collection. GB participated in the design of the study and supervised the work. All authors read and approved the final manuscript.
